# Transition Readiness of Pediatric Sickle Cell Patients to Adult Clinic in a Teaching Hospital, Ghana

**DOI:** 10.1155/ah/2843974

**Published:** 2025-07-10

**Authors:** Aaron Kwasi Nartey, Vivian Paintsil, Isaac Nyanor, Yaa Gyamfua Oppong-Mensah, Evans Xorse Amuzu, Eunice Agyeman Ahmed, Suraj Yawnumah Abubakar, Alex Osei-Akoto

**Affiliations:** ^1^Kumasi Center for Sickle Cell Disease (KCSCD), Komfo Anokye Teaching Hospital, Kumasi, Ghana; ^2^Sickle Cell Unit, Komfo Anokye Teaching Hospital, Kumasi, Ghana; ^3^Directorate of Child Health, Komfo Anokye Teaching Hospital, Kumasi, Ghana; ^4^Department of Child Health, School of Medicine and Dentistry, Kwame Nkrumah University of Science and Technology, Kumasi, Ghana; ^5^Laboratory Services Directorate, Komfo Anokye Teaching Hospital, Kumasi, Ghana

**Keywords:** adolescent, hematology, Kumasi-Ghana, sickle cell disease, transition readiness, young adult

## Abstract

**Background:** Successfully navigating the transition process has received little attention, especially in sub-Saharan Africa. This study assessed the transition readiness of pediatric sickle cell disease (SCD) patients in the Komfo Anokye Teaching Hospital (KATH), Kumasi-Ghana.

**Methods:** A hospital-based cross-sectional study was conducted using a purposive sampling technique to recruit adolescents who were scheduled to be transitioned from the Pediatric to the Adult SCD Clinic at KATH. Two transition assessment tools were adopted and modified to suit our local setting.

**Findings:** Majority of the patients (90%) scored above median mark for the items under the transition self-care importance and confidence and over 50% for most of the items under the disease knowledge and appointment keeping domains. The internal consistencies of the items were over 70% for all the three domains: disease knowledge, medication management, and appointment keeping. In multivariable regression models, older age, female gender, and higher education were associated with higher scores in all the three domains. Also, the sickle cell disease-SS (SCD-SS) status was associated with higher scores in disease knowledge and appointment keeping. Patients staying with both parents were associated with higher scores for the domains but only appointment keeping was statistically significant. Staying with other relations was associated with a lower score for appointment keeping and had significant association for medication management.

**Conclusion:** The study revealed a high transition readiness among pediatric patients. In general, the patients had high confidence transitioning to an adult clinic and the ability to manage their own healthcare. However, patients were hesitant speaking about their SCD status. Staying with both parents was significantly associated with higher scores for appointment keeping. Also, staying with other relations significantly reduced the scores for medication management. We recommend setting up of an adolescent sickle cell support group to help reduce stigmatization and improve health outcomes.

## 1. Introduction

Sickle cell disease (SCD) is a major public health problem plaguing the nations of the world [1, 2]. Globally, it is estimated that 305,800 children are born every year with SCD, with almost 75% of these births occurring in sub-Saharan Africa [3]. In Ghana, it is estimated that 2% of newborns have SCD [4].

The World Health Organization prescribes awareness creation, improvement in disease prevention, and early detection in Africa [5, 6] to curb the menace. The Sickle Cell Unit of the Komfo Anokye Teaching Hospital, Ghana, started championing awareness creation, prevention of SCD, and newborn screening to aid early detection and treatment of children with SCD in 1993 [7]. This increased attention to SCD is aimed at reducing sickle cell-related morbidity and mortality while improving care.

Data show that more than 95% of children born with SCD in UK and USA are surviving into adulthood [8] with life expectancy being estimated as 45–55 years of age [9]. However, Grosse et al., 2011, revealed that death among children born with sickle cell anemia in sub-Saharan Africa is high as 90% in rural communities where there are limited access to healthcare but 50% in populations with lower exposure to infectious diseases and better access to healthcare [10].

Generally speaking, there has been an increase in the survival rate of adolescents with SCD and other chronic conditions, which has resulted in most adolescents growing into adulthood over the past decades [11, 12]. This could also be as a result of improvements in the awareness and management of SCD [11, 13, 14]. In the past, children born with SCD were unlikely to survive into adulthood but with the advancement in medical care, most children with SCD now survive into adulthood [15]. There is, therefore, an increasing need to transition adolescents from the pediatric to the adult-focused care for continuity of quality healthcare.

Also, the pediatric and adult sickle cell clinics established a transition clinic for the adolescents with SCD who needed to be transferred to the adult clinic at Komfo Anokye Teaching Hospital (KATH). There was thus the need to assess their readiness for the transition.

Transition was defined by Major and Hoehner as the purposeful, planned movement of young adults and adolescents with chronic medical and physical conditions from a child-centered care system to an adult-centered health care system [16]. For a transition to be successful, there is the need for a strong collaboration between the patient (young adult/adolescent), the healthcare system, and the caregivers/family [17]. This is needed to support the patient to build skills for independent living and positive disease self-management [16]. It has been advised that planning for transition should start early with disease-specific knowledge. Alashkar et al. recommended that health professionals should offer appropriate participatory training and discussion sessions for patients and their caregivers/parents as early as age 14 [18]. The transition is proposed to comprise psychosocial, educational, and medical needs [19]. In a developed country like the USA, transition from pediatric to adult clinic occurs between the ages of 18 and 21 years. On the other hand, transition in countries in sub-Saharan Africa like Ghana occurs when patients are in their mid-teens [20].

In sub-Saharan Africa and other parts of the world, transitioning from pediatric-centered care to adult-centered clinic is fraught with challenges including lack/inadequacy of adult-care centered facilities and clear-cut responsibilities of the three main actors in the transition process which includes patients, health workers, and caregivers [21]. Successfully navigating the transition process has received little attention despite its significance in caring for SCD patients [13], and this has created a challenge for the stakeholders in the transition process.

In light of this, this study used a quantitative approach to assess the transition readiness of pediatric sickle cell patients in the KATH, Kumasi-Ghana.

## 2. Methods

### 2.1. Study Setting

The study was carried out at the Kumasi Centre for Sickle Cell Disease (KC-SCD) of KATH. KATH is the second largest tertiary referral hospital in Ghana. The KC-SCD is one of the two largest established specialized SCD centers in Ghana. Patients are routinely enrolled into the pediatric clinic from the newborn screening program which started in 1993 [7]. Referrals of patients diagnosed after presenting with complications are also received by the center. A team of hematologists, resident doctors, and nurses provide the clinic's large number of patients with standardized SCD care. About 150 pediatric patients and 30 adult patients with SCD are attended to every week. Pediatric patients with SCD are routinely referred to the adult SCD clinic by age 14 years.

Prior to transition of pediatric patients, health education talks are routinely provided to improve understanding of the disease processes and management measures. The most prevalent SCD variants in our center are sickle cell disease-SS (SCD-SS) and sickle cell disease-SC (SCD-SC) [7, 22].

### 2.2. Research Design

A hospital-based cross-sectional study was conducted using a purposive sampling method; 38 adolescents who were scheduled to be transitioned from pediatric to the adult-centered clinic of the facility were interviewed with a modified Transition Readiness Assessment Questionnaire (TRAQ).

### 2.3. Sampling Strategy

A total of three [3] transition clinics were held in 2021 for adolescents who met the age of transition (14 years and above). These transition clinics were joint clinics of the adult and pediatric hematologists and their nurses. These joint clinics were held every four [4] months as a final clinic for the adolescent with SCD before they were transitioned to the adult clinic. The study lasted for one year, January–December 2021. A total of 38 adolescents were transitioned to the adult-centered care. All the patients and their caregivers who were approached to participate in the study complied.

### 2.4. Transition Clinic

KATH transition clinic is a joint clinic of adult and pediatric hematologists and their nurses, which is held fourth monthly for adolescents who are of age (14 years and above). Adolescent SCD patients could attend one or multiple transition clinic(s) before transitioning to the adult clinic. Patients who score above or equal to the minimum required scores and have no clinical complications are deemed ready for transition to an adult clinic whereas patients who score below the required mark are made to attend subsequent transition clinics till they are well prepared for an adult clinic.

During transition clinics, patients are taken through a discussion on the disease, pattern of inheritance, health maintenance with emphasis placed on clinic attendance, and medication compliance, as well as reproductive health. The education sessions are usually interactive and participants are encouraged to contribute and ask questions. With both the pediatrician and adult hematologist present, each patient is seen and any peculiarities were shared for improved continuity of care.

### 2.5. Data Collection Tool

Two transition assessment tools were adopted and modified to meet the needs of our local setting. These transition assessment tools were the Sickle Cell Disease Transition Readiness Assessment Template of the American Society of Hematology (American Society of Hematology, 2016) [23] and TRAQ [24]. These two transition readiness assessment templates have been validated in similar populations in the USA [18].

The Sickle Cell Disease Transition Readiness Assessment Template of the American Society of Hematology is a tool used to assess transition readiness to adult clinics in both adolescents and young adult sickle cell patients. This tool was developed to assess skills, self-care knowledge, and additional issues that need to be addressed to ensure optimal management of their health conditions (American Academy of Pediatrics, American College of Physicians, American Society of Hematology, 2016). The TRAQ is a validated, patient-centered questionnaire used to assess a youth or adolescent's ability to make appointments, understand their medications, and develop other skills needed for transition to adult care [25]. TRAQ can be used by child health providers, family members, and educators to identify areas in which adolescents and youths need training and education to achieve independence in transition-relevant skills [25].

Our question merged the transition and self-care importance and confidence, disease knowledge, medication management, and appointment domains of the questionnaire from the American Society of Hematology's Sickle Cell Disease Transition Readiness Assessment Template and the TRAQ, modifying it appropriately to fit the local setting of the study area.

The adopted templates helped in the development of a local toolkit that was fit for purpose and met the requirements of our local setting. The American Society of Hematology Sickle Cell Disease Transition Readiness Assessment Template has five main domains under “My Health;” these include disease knowledge, medication management, appointment, insurance, and privacy knowledge, with each of these domains having specific questions under them. The first three domains (disease knowledge, medication management, and appointment) were adopted for this study [26]. Under medication management, the question “I am aware of what hydroxyurea is and how it prevents sickling of my red blood cells” and “I know how to get medical care when the doctor's office is closed” under appointment were also dropped because most of the participants were not taking hydroxyurea and do not know what it is.

Also, the responses provided on the TRAQ were adopted for the “My Health Responses” of the study. These responses include the following: No, I do not know how; No, but I want to learn; No, but I am learning to do this; Yes, I have started doing this; and Yes, I always do this when I need to [25].

### 2.6. Scoring

The scoring for this work has been explained in Tables 1 and 2.

The Transition Self-Care Importance and Confidence scale was scored with marks greater or equal to five [5] as indication for having confidence in self-care and marks lower than five [5] not having confidence. The categorization of the scale was made with the median mark.

These scores were used for all the items and respondents were expected to obtain an overall average of 3.0 to be considered adequate for a specific domain. The highest score was set at 5 and scoring less than 3.0 was indicative of unpreparedness for transition while any score 3.0 and above was indicative of preparedness for transition.

## 3. Data Collection, Management, and Analysis

An electronic questionnaire was designed with Research Electronic Data Capture (REDCap) Version 13.8.2 [27] and setup on a tablet using the REDCap mobile app for the data collection [28]. Trained research assistants administered the questionnaires to patients while they waited for the transition clinic to start.

The data were exported to Stata/SE 17.0 Statistical Software (StataCorp. 4905 Lakeway Drive College Station, Texas 77,845, USA) for cleaning and analysis. The reliability of the test items was assessed with Cronbach's alpha. A Cronbach's alpha of 0.70 (70%) or higher was considered acceptable [29]. Categorical variables were organized into frequencies and percentages. Continuous variables were summarized by mean and standard deviation (SD) or median and interquartile range (IQR) where appropriate. The summated scale was used, and means with standard deviations were calculated from the results. The ordinal scale data were assumed to have equality of intervals. The Chi-squared test of association and multivariable regression analysis were used to compare the three domains: disease knowledge, medication management and appointment keeping, and demographic characteristics. A *p* value of < 0.05 was considered statistically significant.

### 3.1. Ethical Consideration

Ethical approval was obtained from the Committee on Human Research Publication and Ethics, a joint committee of the KNUST School of Medical Sciences (KSMD) and KATH with Protocol number: CHRPE/AP/033/21. Informed consent was secured from each person deciding to respond to the questionnaire. For those less than 18 years, consents were sought from caregivers/family while patient assent was also taken from the participants.

## 4. Results

### 4.1. Demographic Characteristics of the Respondents

The mean age of the patients was 17.45 years with a standard deviation of 1.52 years. There was an equal number of males and females with majority (68.42%) currently at the Senior High School level. Most of the patients (81.50%) were SCD-SS phenotype and were diagnosed of SCD as newborns (71.05%).  Table 3 gives the detailed demographic characteristics of the respondents.

## 5. Transition Readiness Assessment

### 5.1. Transition Self-Care Importance and Confidence

In assessing the transition self-care importance and confidence of the patients, three questions were asked: “How important is it to you to manage your own health,” “How confident do you feel about your ability to manage your own health care,” and “How confident do you feel about preparing for/changing to an adult doctor,” and patients scored more than 90%. Details on the importance of self-care and respondents' confidence are provided in Table 4.

### 5.2. My Health Response

Patients scored over 50 percent for all the items under the disease knowledge domain except for the following three items: I have friends that I can talk to about SCD, (26.32%), I know about necessary screening exams (echo, kidney function retinal exams, etc.) (47.37%), and I know how to get blood work and x-rays (44.74%).

Items under medication management scored more than 60% while items under appointment also scored more than 50% with exception “I make my own doctor's appointments,” which patients scored 47.37%.  Table 5 gives details about My Health Responses.

### 5.3. Relationship Between Demographic Characteristics and Participants Phenotype

We assessed whether there was any relationship between phenotype and demographic characteristics. Only gender was found to be statistically significant (*p*=0.036) among SCD-SS and SCD-SC patients. In assessing the health of the patients (in terms of disease knowledge, medication management, and appointment), patients with SCD-SS scored higher in terms of appointment and medication management, but there was no significant relationship noted. There was, however, a statistically significant relationship (*p*=0.015) between the SCD-SS phenotype and appointment keeping.  Table 6 gives details about the relationship between demographic characteristics and patient phenotype.

### 5.4. Multivariable Regression Analysis of Disease Knowledge and Demographic Characteristics

A unit increase in age is associated with an average increase in score of 0.17 of disease knowledge in the bivariable analysis, and taking the effect of all other variables into consideration at the multivariable stage, it reduced to 0.11 but none was statistically significant. Females scored 0.29 higher than their average male counterparts. While holding all the other variables constant, the score dropped to 0.26 (statistically insignificant). Higher educational level than JHS of patients is associated with a higher score in disease knowledge but the score decreased when all the other variables were held constant (SHS: 0.79–0.58 and Tertiary: 1.39–0.94). Patients with SCD-SS phenotype scored 0.17 higher on average than patients with SCD-SC phenotype in disease knowledge and holding all other variables constant SCD-SS had an average score of 0.24 higher than patients with SCD-SC phenotype. Patients who stayed with both parents scored 0.40 on average higher than those staying with their mothers. Also, those staying with “Other” recorded decrease of 0.87 than those staying with their mothers; details are presented in Table 7.

### 5.5. Multivariable Regression Analysis of Medication Management and Demographic Characteristics

A unit increase in age is associated with an increase in average score of 0.11 in medication management, and holding other variables constant, it reduced to 0.08 but none was significant. Females scored higher than males (0.01), and this increased to 0.05 after holding all other variables constant but the differences were not statistically significant. The higher the educational level of patients, the higher the score; the same was again witnessed when all the other variables were held constant (SHS: 0.38–0.15 and Tertiary: 0.46–0.02). Patients with SCD-SS phenotype scored 0.03 higher on average than patients with SCD-SC phenotype in medication management, and holding all other variables constant, SCD-SS phenotype scored lower (−0.01) than participants with phenotype SCD-SC in medication management. Patients who stay with both parents scored 0.22 on average higher than those staying with their mothers. Also, those staying with “other relation” scored lower (−1.14) than those staying with their mothers.  Table 8 presents the regression analysis of medication management and demographic characteristics of adolescent SCD patients.

### 5.6. Multivariable Regression Analysis of Appointment and Demographic Characteristics

A unit increase in age is associated with an average of 0.16 higher score in appointment keeping. Holding other variables constant, the score was reduced to 0.12. Females scored less than their male counterparts (−0.21), and holding all other variables constant female scored on average 0.01 more than males. The higher the educational level of patients, the higher the score on appointment keeping; the same was witnessed when all the other variables were held constant (SHS: 0.02–0.44 and Tertiary: −0.33–0.61). Patients with SCD-SS phenotype scored 0.74 on average than patients with SCD-SC, and holding all other variables constant, SCD-SS scored 0.52.

Patients who stay with both parents scored 0.77 on average higher than those staying with their mothers alone. Also, those staying with “Other” scored lower (−1.02) than those staying with their mothers. Holding all other variables constant patients staying with “Both Parents” scored 0.67 while that of “Other” reduced to −1.01. Details of the regression analysis of appointment and demographic characteristics of adolescent SCD patients are present in Table 9.

### 5.7. Summated Mean Scores

The summated mean for disease knowledge was 4.00, for medication management was 4.63, and for appointment keeping was 4.36. These scores for the three domains revealed that all patients were well qualified to be transitioned to adult-centered care since the summated means were above 3.00.

Cronbach alpha was used to measure the reliability of the test of which a minimum of 0.70 (70%) is required to meet the reliability test. The reliability of disease knowledge of the patients was assessed with a reported Cronbach alpha of 0.8921 (89%). Also, a Cronbach alpha of 0.729 (73%) and 0.8396 (84%) was scored for medication management and appointment keeping, respectively.  Table 10 presents the summated mean scores of My Health for Adolescent SCD patients.

## 6. Discussion

Mortality among sickle cell patients in developing countries, especially Africa, was high until recently when improvement in medical care has helped in reducing it [11, 20, 31]. With the emergence of newborn screening program for SCD at KATH and the introduction of special disease care, many SCD patients are surviving into adulthood [11, 17], which necessitates the transition of adolescent from pediatric clinic to adult-centered care. At the KC-SCD-KATH, 38 SCD patients who were scheduled to be transitioned from the pediatric clinic to adult-centered care in 2021 had their readiness assessed. The focus of this transition readiness was centered on three domains: disease knowledge, medication management, and appointment keeping.

### 6.1. Sociodemographic Characteristics of Patients

The sociodemographic characteristics of the study patients showed that the number of males were the same as the number of females. This finding is consistent with studies by Stephen et al. [32], Laurence et al. [33] Hair et al. [29]. Again, the finding agrees with the results of a study by Asnani et al., which revealed that sickle cell affects males and females equally because the inheritance is autosomal recessive [32]. However, a study by Paintsil et al. in the same site revealed a higher percentage of males SCD patients [7]. With regards to educational level, more than half of the patients had secondary education compare to having Junior High level of education, this could be associated to the kind of participants engaged (adolescents). A study by Kwarteng-Siaw et al. on the assessment of transition readiness in adolescents with SCD revealed contrary findings: 61% were at the Junior High School while 41% with secondary education [20]. Another study by Laurence et al. is consistent with our findings, which had majority of the patients having the secondary school level of education [33].

Majority of the patients who were being transitioned had SCD-SS phenotype. This result is consistent with Kwarteng-Siaw et al., which showed more SCD-SS phenotype in their study compared with SCD-SC patients [30]. Also, Paintsil et al. showed that more patients with SCD-SS phenotype are seen at the clinic as compared to those with SCD-SC [7]. This can be attributed to the fact that patients with SCD-SS phenotype experience the severe form of the disease than SCD-SC and, therefore, tend to report to clinic more frequently.

### 6.2. Transition Self-Care Importance and Confidence

The study assessed the importance of transition to the pediatric SCD patients as well as their confidence levels in moving them to the adult clinic. In general, the transition self-care importance and confidence of the pediatric patients were very high. Majority of the patients scored high marks, which indicates that managing their own health was important to them. On assessing how important it is to manage their own health, most of the patients scored high marks. Almost all patients who participated in the study had high scores on how confident they feel about preparing for/changing to an adult doctor. This revealed that self-care is very important for adolescent sickle cell patients at the KC-SCD clinic. This corroborates with a study by Nagshabandi and Abdulmutalib in Saudi Arabia, which revealed that self-care activities and self-care capability among sickle cell patients were high [31]. The patients were seen to be knowledgeable, and this could be attributed to the level of education of the patients. Self-care activities among sickle cell patients contribute to good health and wellbeing, and patient may need fewer hospital appointments. These findings also coincide with Edwards et al. and Matthie et al., which revealed higher level of education and SCD self-efficacy playing an important role in the self-care of SCD patients [35, 36]. The finding is also consistent with Sobota et al. who revealed that persons living with SCD have knowledge about the disease and know it; they become very confident to face challenges in self-care [37]. This high score could also be as a result of the pretransition education, which empowers adolescents to be able to undertake basic self-care activities.

### 6.3. Disease Knowledge

For an adolescent SCD patient to be successfully transitioned to the adult-centered care, their knowledge on SCD was assessed using the adopted assessment tools (Table 4). Knowledge about SCD is important because transition to adult-centered care begins the period where most of the care management is moved from the caregiver to the patients [16, 21].

The study patients had high disease knowledge. The two items “Yes, I have started doing this” and “Yes, I always do this when I need to” for all the items under disease knowledge had higher scores. The question with the highest score was “I know what type of SCD I have.” On the other hand, an item like “I have friends that I can talk to about SCD” scored less than the other items.

The high responses on disease knowledge could be associated to the level of education among the patients. Their educational level could contribute to a better understanding of educational material given to them thus leading to a high disease knowledge. The second highest item score was “I know my medical needs and can explain them to someone” for the yes responses of “Yes, I have started doing this” and “Yes, I always do this when I need to”. It is expected that transitioned patients should be able to communicate their health issues to anyone as required especially the healthcare providers when they go to the clinic and not to depend on their caregivers. Therefore, patients scoring high for “Yes” on that question means the patients are ready to be transitioned. This finding is in contrast with a study by Faremi and Olawatosin in Nigeria which revealed that majority of the adolescent sickle cell patients had poor knowledge of the disease [38], which also confirms a submission by Acharya et al., which reported low level of knowledge among adolescent sickle cell patients in Nigeria which could be attributed to their level of education [39]. This study reveals high adolescent SCD patients readiness for transition as opposed to a study carried out in the same hospital by Kwarteng-Siaw et al. [20]. This is due the specialized care provided by both pediatric and adult hematologist that is now available in the clinic. This helps to make sure that the patient feels welcome and ready for transition.

The item “I have friends that I can talk to about SCD” had the lowest score. This might be attributed to the effect of stigmatization. Adolescent sickle cell patients would not want others especially friends, classmates, and teachers to know their status because of stigma. Most often, the general public is not educated and sensitized on SCD. They hold on to their own misconception about the disease which is typically negative, which leads to stigmatization against people living with SCD [40, 41]. This confirms a study by Ola et al., which revealed that individuals with SCD reported experiencing stigma or negative reaction from friends/family, community members, coworkers, and peers regarding their SCD status [42]. Stigmatizing of person living with SCD results to them being devalued and experiencing status loss. They also have their pain experiences discredited by others and accused of being lazy, weak or pretending to be ill, pitied, or treated differently [40, 42]. SCD patients would prefer not to disclose their status to prevent the anxiety, anticipated stigma, or stigma [42].

### 6.4. Medication Management

Medications over the period have been developed to improve the duration and quality of patient's lives [43], leading to medication adherence becoming fundamentally the self-management of patients condition [44]. Management of sickle cell anemia is generally aimed at reducing or avoiding pain episode, preventing complication, and relieving symptoms.

The reliability of the questions under medication management was checked by Cronbach alpha, which indicated an acceptable reliability. Medication management had a Cronbach alpha of 0.729 (73%), greater than the minimum score of 70%. Also, medication management had a summated mean of 4.63.

The study assessed the medication management among the participants since it is very important in the disease management. The patients had a good score for the medication management practice. In summing up the two “Yes” responses (“Yes, I have started doing this” and “Yes, I always do this when I need to”), patient scored high percentages in all the items. Two items “I know the names and doses of my medications” and “I remember to take my medications without my parents reminding me” had a high score. This implies that the medication adherence level of patients is very high. This confirms the findings from Kinney et al., 1999, and Thornburg et al., 2010, which revealed as high as 94% medication adherence among adolescents [45, 46].

However, a systematic review by Curtis et al. on adherence to medication by sickle cell patients revealed moderate adherence to medication by adolescents [44]. Poor adherence to medication leads to increase susceptibility to complication, reduced effectiveness of medication, and medical wastage [47, 48]. It is, therefore, necessary to assess the medication management among sickle cell patients before their transition into adult-centered clinic.

The lowest percentage score within the medication management was for the item “I know what my medications are for.” Although it is a moderate percentage, being the lowest among the set of questions can be attributed to the fact that, healthcare providers (doctors and nurses) might not have explained the specific medications and the reason for them taking it or their caregivers who would know the use might not have explained to some of these adolescents.

### 6.5. Appointment Keeping

Sickle cell guidelines recommend that patients attend routine clinic appointment to help monitor their health and wellbeing [49]. Medical advances including chronic transfusion [50] and use of hydroxyurea [51] have resulted to improved treatments for adolescents with SCD [52–54]. However, patients' access to pharmacological and medical therapies is dependent on routine care, follow-up on hematologists who refer patients to laboratory services, medication refill, monitoring of side effects and benefits of treatment [49], and x-ray when the need be.

The reliability of the questions under appointment keeping was checked by Cronbach alpha which indicated an acceptable reliability. Appointment keeping had a Cronbach alpha of 0.8396 (84%), greater than the minimum score of 70%. Again, appointment keeping had a summated mean of 4.36.

In summing up the two “Yes” responses (“Yes, I have started doing this” and “Yes, I always do this when I need to”), the study revealed the attitude of participants towards appointments. Patients had a high score for the item “I answer questions on my own during medical visit,” which happened to be the highest score from the questions under the “appointment keeping.” This indicates that adolescent patients about to be transitioned can attend clinic, present their challenges, and answer questions from physicians without assistance from their caregivers. The second highest score was the question “I keep track of my doctor's and other appointments.” This reveals that the patients would be able to know and keep the date of visits for their routine care. About 85% of the patients responded “Yes” for the item “I follow-up on any referral for test, check-up, or lab” and “I keep a calendar or list of medical or other appointments.” These positive responses from the study patients confirm the study by Sobota et al., which looked into self-reported transition readiness among young adult with SCD, which also revealed 85% of the patients reporting they knew how to make their own appointments [11]. A majority (91%) of the patients revealed they could get their prescription medications filled on their own. However, fewer (79%) indicated that they go to their doctor's appointment alone, which also confirms that 71.05% of them, indicating they arrange their own transport to medical appointments. This revealed that those who come with caregivers depend on them for transport arrangement. Although these percentages (79% and 71.05%) are quite high, they are among the lowest scores from the respondent. This confirms a study by Crosby et al. carried out in the USA, which sought to find out the perceived barriers to clinic appointments for adolescents with SCD [49]. The study revealed one major factor that influences adolescent sickle cell patient's ability to make it to doctor's appointment. They reported that the patients are more likely to attend clinic appointment if their caregivers/parents were involved.

### 6.6. Multivariable Regression Analysis of Demographic Characteristics and the Three Domains

The relationship between demographic factors and the three domains were assessed in regression analyses. Older age, female gender, higher education, SCD-SS status, and staying with both parents were associated with higher scores for the disease knowledge, medication management, and appointment keeping domains. However, none of these variables were significantly associated with these domains. As expected, older age was associated with higher scores in these domains. This suggests that as adolescents SCD patient get older, they gain understanding of their condition through self-learning. They progress along a path of skilled-building in terms of disease knowledge, medication management, and appointment keeping. However, age per se was not a relevant predictor of these domains. This finding is in line with what was observed in a study, which found that older age was associated with higher scores in the self-management skills [55]. Our result suggests gender differences exist in the scores of the domains. Females scored higher than their males' counterpart. This might be due to gender differences in adolescent maturity or perhaps due to societal expectations as reported by Sawicki et al. [55]. Girls are reported to achieve developmental milestones such as marriage, living independently, and having children earlier than boys [56]. Also, a study reported young women had higher relational maturity and expectations in terms of emerging adult skills [57]. Our study reveals higher education status increases scores for disease knowledge and medication management domains. However, appointment keeping domain has a higher score for only senior high school SCD patients, but this effect is lost as they progress through a tertiary education. This might be due to busy schedules and challenging academic duties that are associated with higher education. The linkage of education to skill development has been shown in other studies to contribute to independent living and mastery of skills related to disease self-management and healthcare utilization [58, 59]. SCD-SS patients tend to score high for disease knowledge and appointment keeping domains. However, a lower score was observed for medication management for this same group. SCD-SS patients have the severe form of the disease and are more likely to report at hospital regularly [60, 60]. The encounters with health practitioners build their capacity in terms of disease knowledge and appointment keeping. However, they had lower scores for medication management. This might be due low adherence which this study could not ascertain.

Limitations of this study include its modest sample size at a single SCD clinic. Hence, the results may not be representative of transition at other SCD clinics in Ghana or elsewhere in the region. The cross-sectional nature of this study only gives an indication of the potential association of factors for a health outcome and cannot prove causation. However, cross-sectional studies such as ours can help in generating research hypothesis.

## 7. Conclusion

The study revealed the high level of transition readiness among the patients. Patients who stay with both parents, increase age, being a female, higher education, and SCD-SS status had a higher score on average for all the domains, an indication of their readiness for transition. Staying with both parents was significantly associated with higher scores for appointment keeping. Also, staying with other relations significantly reduced the scores for medication management. In general, the patients were highly confident about preparing for/changing to an adult doctor, and also, they were equally confident about their ability to manage their own healthcare. However, patients do not have friends to talk to about the disease due to the fear of being stigmatized.

We, therefore, recommend setting up of adolescent transition clinic across sub-Saharan Africa to encourage those within the transition age to attend clinic regularly for better health outcome. Also, an adolescent sickle cell support group should be set up to help reduce stigmatization.

## Figures and Tables

**Table 1:** Response for transition and self-care importance and confidence (a scale of 0–10).

Item	Score category
How important is it to you to manage your own health	< 5
	≥ 5

How confident do you feel about your ability to manage your own healthcare	< 5
	≥ 5

How confident do you feel about preparing for/changing to an adult doctor	< 5
	≥ 5

*Note:* < 5: not confident. ≥ 5: confident.

**Table 2:** Response for individual item scores on my health.

Response	Scores
No, do not know how	1
No, but I want to learn	2
No, but I am learning to do this	3
Yes, I have started doing this	4
Yes, I always do this when I need to	5

**Table 3:** Demographic characteristics of adolescent SCD patients.

Variable, *N* = 38	Frequency	Percentage
Gender		
Male	19	50.00
Female	19	50.00
Age in years		
Mean (±SD) = 17.45 (±1.52)		
Phenotype		
SS	31	81.50
SC	7	18.42
How did you test for SCD		
Newborn screening	27	71.05
Nonnewborn screening	11	28.95
Educational level		
JHS	10	26.32
SHS	26	68.42
Tertiary	2	5.26
Student		
Yes	35	92.11
No	3	7.89
If yes, what is your school residential status [30]		
Residential	15	42.86
Nonresidential	20	57.14
Number of siblings, median (IQR)	3 (2–4) children	
Number of siblings with SCD		
None	24	63.16
1	9	23.68
2	2	5.26
3	3	7.89
Caregiver		
Mother	12	31.58
Both parents	25	65.79
Other	1	2.63

Abbreviations: IQR = interquartile range, JHS = Junior High School, SCD = sickle cell disease, SD = standard deviation, SHS = Senior High School.

**Table 4:** Transition self-care importance and confidence of adolescent SCD patients.

Variable, *N* = 38	Scoring categories	Frequency	Percentage
How important is it to you to manage your own health	< 5	3	7.89
	≥ 5	35	92.11

How confident do you feel about your ability to manage your own healthcare	< 5	2	5.26
	≥ 5	36	94.74

How confident do you feel about preparing for/changing to an adult doctor	< 5	1	2.63
	≥ 5	37	97.37

**Table 5:** My Health Responses.

Questionnaire item, *N* = 38	No, I do not know how	No, but I want to learn	No, but I am learning to do this	Yes, I have started doing this	Yes, I always do this when I need to
*Disease Knowledge, n (%)*					
I know what type of sickle cell disease I have	2 (5.26)	1 (2.63)	0 (0.00)	5 (13.16)	30 (78.95)
I know my medical needs and can explain them to someone	3 (7.89)	2 (5.26)	1 (2.63)	4 (10.53)	28 (73.68)
I know what a hematologist is and why I go to one	3 (7.89)	4 (10.53)	4 (10.53)	4 (10.53)	23 (60.53)
I know what to do in case of medical emergency	3 (7.89)	4 (10.53)	1 (2.63)	5 (13.16)	25 (65.79)
I understand what causes a pain episode	2 (5.26)	3 (7.89)	1 (2.63)	6 (15.79)	26 (68.42)
I understand how drugs, alcohol, and tobacco affect sickle cell disease	2 (5.26)	6 (15.79)	3 (7.89)	7 (18.42)	20 (52.63)
I have friends that I can talk to about sickle cell disease	14 (36.84)	2 (5.26)	2 (5.26)	10 (26.32)	10 (26.32)
I know about necessary screening exams (echo, kidney function retinal exams, etc.)	3 (7.89)	5 (13.16)	6 (15.79)	6 (15.79)	18 (47.37)
I know how to get blood work and x-rays.	3 (7.89)	8 (21.05)	4 (10.53)	6 (15.79)	17 (44.74)

*Medication Management, n (%)*					
I know what my medications are for	0 (0.00)	2 (5.26)	1 (2.63)	5 (13.16)	30 (78.95)
I know the names and doses of my medications	0 (0.00)	1 (2.63)	0 (0.00)	4 (10.53)	33 (86.84)
I remember to take my medications without my parents reminding me	0 (0.00)	1 (2.63)	0 (0.00)	7 (18.42)	30 (78.95)
I fill prescriptions before I run out of medications	2 (5.26)	0 (0.00)	5 (13.16)	7 (18.42)	24 (63.16)
I know how to prevent a pain episode and what to do if I have pain	0 (0.00)	3 (7.89)	1 (2.63)	7 (18.42)	27 (71.05)

*Appointments Keeping, n (%)*					
I make my own doctor's appointments	3 (7.89)	3 (7.89)	4 (10.53)	10 (26.32)	18 (47.37)
I keep track of my own medical information	1 (2.63)	2 (5.26)	3 (7.89)	5 (13.16)	27 (71.05)
I keep track of my doctor's and other appointments	1 (2.63)	0 (0.00)	2 (5.26)	8 (21.05)	27 (71.05)
I answer questions on my own during medical visit	0 (0.00)	0 (0.00)	1 (2.63)	8 (21.05)	29 (76.32)
Do you arrange your own transport to medical appointments	3 (7.89)	1 (2.63)	7 (18.42)	5 (13.16)	22 (57.89)
I follow-up on any referral for test, check-up, or lab	1 (2.63)	1 (2.63)	5 (13.16)	6 (15.79)	25 (65.79)
I keep a calendar or list of medical or other appointments	3 (7.89)	1 (2.63)	3 (7.89)	6 (15.79)	25 (65.79)

**Table 6:** Relationship between demographic characteristics and patient phenotype.

Factor, *N* = 38	Phenotype, *n* (%)		*p* value
	SS, *n* = 31	SC, *n* = 7	
Age in years	17.58 (1.61)	16.86 (0.90)	0.260
Gender			
Male	18 (94.74)	1 (5.26)	0.036
Female	13 (68.42)	6 (31.58)	
Education level			
JHS	8 (80.00)	2 (20.00)	1.000^∗^
SHS	21 (80.77)	5 (19.23)	
Tertiary	2 (100.00)	0 (0.00)	
Caregiver			
Mother	8 (66.67)	4 (33.33)	0.333^∗^
Both parents	22 (88.00)	3 (12.00)	
Other	1 (100.00)	0 (0.00)	
Assessment of Health, mean (±SD) score			
Disease Knowledge	3.97 (1.01)	4.14 (0.83)	0.674
Medication Management	4.63 (0.52)	4.60 (0.42)	0.880
Appointment keeping	4.50 (0.63)	3.76 (0.95)	0.015

^∗^Fisher's exact test was performed because of limited cell count.

**Table 7:** Multivariable regression analysis of disease knowledge and demographic characteristics.

Variable	Disease knowledge					
	Unadjusted			Adjusted		
	Coeff.	95% CI	*p* value	Coeff.	95% CI	*p* value
Age	0.17	−0.04–0.38	0.104	0.11	−0.13–0.34	0.359
Sex						
Male	Ref					
Female	0.29	−0.35–0.93	0.363	0.26	−0.43–0.95	0.448
Education						
JHS	Ref					
SHS	0.79	0.09–1.48	0.027	0.58	−0.22–1.37	0.150
Tertiary	1.39	−0.54–2.83	0.059	0.94	−0.75–2.63	0.264
Phenotype						
SC	Ref					
SS	0.17	−1.01–0.66	0.674	0.24	−1.17–0.69	0.603
Caregiver						
Mother	Ref					
Both parents	0.40	−0.29–1.09	0.245	0.27	−0.48–1.01	0.467
Other	−0.87	−2.91–1.17	0.392	−0.43	−2.59–1.73	0.687

*Note:* Coeff: coefficient.

Abbreviations: CI = confidence interval, JHS = Junior High School, SHS = Senior High School.

**Table 8:** Regression analysis of medication management and demographic characteristics of adolescent SCD patients.

Variable	Medication management					
	Unadjusted			Adjusted		
	Coeff	95% CI	*p* value	Coeff	95% CI	*p* value
Age	0.11	0.001–0.21	0.047	0.08	−0.03–0.19	0.158
Sex						
Male	Ref					
Female	0.01	−0.34–0.32	0.949	0.05	−0.27–0.38	0.751
Education						
JHS	Ref					
SHS	0.38	0.02–0.75	0.040	0.15	−0.23–0.53	0.426
Tertiary	0.46	−0.29–1.22	0.227	0.02	−0.78–0.82	0.953
Phenotype						
SC	Ref					
SS	0.03	−0.39–0.46	0.880	−0.01	−0.45–0.43	0.948
Caregiver						
Mother	Ref					
Both parents	0.22	−0.09–0.53	0.166	0.19	−0.16–0.54	0.288
Other	−1.32	−2.25–−0.38	0.007	−1.14	−2.16–−0.11	0.031

*Note:* Coeff: coefficient.

Abbreviations: CI = confidence interval, JHS = Junior High School, SHS = Senior High School.

**Table 9:** Regression analysis of appointment and demographic characteristics of adolescent SCD patients.

Variable	Appointment keeping					
	Unadjusted			Adjusted		
	Coeff	95% CI	*p* value	Coeff	95% CI	*p* value
Age	0.16	0.01–0.32	0.038	0.12	−0.02–0.27	0.092
Gender						
Male	Ref					
Female	−0.21	−0.70–0.28	0.391	0.01	−0.42–0.44	0.965
Education						
JHS	Ref					
SHS	0.44	−0.12–0.99	0.118	0.02	−0.47–0.51	0.934
Tertiary	0.61	−0.54–1.77	0.289	−0.33	−1.37–0.71	0.525
Phenotype						
SC	Ref					
SS	0.74	0.15 to 1.33	0.015	0.52	−0.05 to 1.09	0.073
Caregiver						
Mother	Ref					
Both parents	0.77	0.33–1.21	0.001	0.67	0.21–1.13	0.006
Other	−1.02	−2.33–0.28	0.121	−1.01	−2.35–0.32	0.132

*Note:* Coeff: coefficient.

Abbreviations: CI = confidence interval, JHS = Junior High School, SHS = Senior High School.

**Table 10:** Summated mean scores of My Health for Adolescent SCD patients.

Domain	Questionnaire item	Mean (±SD)	Cronbach alpha
Disease knowledge			0.8921
	I know what type of sickle cell disease I have	4.58 (1.03)	0.8968
	I know my medical needs and can explain them to someone	4.37 (1.26)	0.8656
	I know what a hematologist is and why I go to one	4.05 (1.37)	0.8597
	I know what to do in case of medical emergency	4.18 (1.35)	0.8739
	I understand what causes a pain episode	4.34 (1.19)	0.8817
	I understand how drugs, alcohol, and tobacco affect sickle cell disease	3.97 (1.33)	0.8644
	I have friends that I can talk to about sickle cell disease	3.00 (1.71)	0.9073
	I know about necessary screening exams (echo, kidney function retinal exams, etc.)	3.82 (1.37)	0.8814
	I know how to get blood work and x-rays.	3.68 (1.44)	0.8839
Total		4.00	

Medication management			0.729 (73%)
	I know what my medication are for	4.66 (0.78)	0.6828
	I know the names and doses of my medications	4.84 (0.44)	0.6292
	I remember to take my medications without my parents reminding me	4.76 (0.49)	0.6575
	I fill prescriptions before I run out of medications	4.34 (1.07)	0.7072
	I know how to prevent a pain episode and what to do if I have pain	4.53 (0.89)	0.7276
Total		4.63	

Appointment keeping			0.8396
	I make my own doctor's appointments	3.97 (1.28)	0.8423
	I keep track of my own medical information	4.45 (1.03)	0.8109
	I keep track of my doctor's and other appointments	4.58 (0.83)	0.7997
	I answer questions on my own during medical visit	4.74 (0.50)	0.8555
	Do you arrange your own transport to medical appointments	4.11 (1.27)	0.8119
	I follow-up on any referral for test, check-up, or lab	4.39 (1.00)	0.7954
	I keep a calendar or list of medical or other appointments	4.29 (1.23)	0.7992
Total		4.36	

## Data Availability

The data used to support the findings of this study are available from the corresponding author upon reasonable request.
